# Evaluation of chatbot and specialist knowledge on pediatric sedation and general anesthesia: a comparative analysis

**DOI:** 10.1186/s12911-026-03748-y

**Published:** 2026-08-05

**Authors:** Dilara Dinç, Aslıhan Ozbilgen

**Affiliations:** Department of Pediatric Dentistry, Faculty of Dentistry, Biruni University, 10. Yıl Street, No: 45 Topkapı,Zeytinburnu, Istanbul, 34010 Turkey

**Keywords:** Artificial intelligence, Large language models, Clinical decision support systems, Pediatric anesthesia, Sedation, Clinical competence

## Abstract

**Background:**

The integration of artificial intelligence (AI) in healthcare has increased rapidly, with large language model-based chatbots emerging as potential tools for education and clinical support. However, their performance in complex medical domains such as sedation and general anesthesia remains underexplored. This study aimed to evaluate the accuracy of AI-supported chatbot models (ChatGPT 4.0 Mini, Gemini 1.5 Pro, and Claude 3 Sonnet) in comparison to human experts (Anesthesiologists, Pediatric Dentists, Oral and Maxillofacial Surgeons) within the context of sedation and general anesthesia.

**Methods:**

This descriptive and comparative cross-sectional study utilized a 26-item true/false questionnaire based on ASA, AAP, ADA, and AAPD guidelines. The questionnaire was administered to three chatbots and 72 human participants (24 per specialty). For chatbot evaluation, a zero-shot prompting technique with a uniform command (“Is this statement true or false?”) was applied in separate sessions after clearing the cache to ensure standardization. Responses were coded as correct/incorrect by two independent pediatric dentists. Human participants completed the same questionnaire via Google Forms. Descriptive statistics were calculated. Normality of data was assessed using the Shapiro-Wilk test. Non-parametric data were compared using the Kruskal-Wallis test followed by Bonferroni-adjusted post hoc tests. Categorical variables were analyzed using the Pearson Chi-square test. Statistical analyses were performed using IBM SPSS v27.

**Results:**

Significant differences were found among all groups ($*p* < 0.001$). In terms of text-based guideline retrieval, Claude 3 Sonnet (92.8%) and Gemini 1.5 Pro (91.7%) demonstrated high factual accuracy, while anesthesiologists achieved the highest performance among human clinicians (87.0%). While the chatbot models showed high proficiency in data retrieval for “General Information” and “Postoperative Monitoring,” anesthesiologists significantly outperformed the AI models in “Patient Evaluation and Preparation” (91.7%), highlighting a critical gap in AI’s clinical reasoning compared to human expertise. Notable knowledge gaps were identified among oral surgeons regarding pharmacological reversal agents.

**Conclusions:**

Claude and Gemini chatbots exhibit high factual accuracy in retrieving guideline-based pediatric sedation theory, suggesting potential exclusively as supplementary informational resources. However, anesthesiologists’ superior performance in pre-operative assessment underscores the irreplaceable role of clinical judgment. AI integration should therefore follow a synergistic model, positioning language models strictly as adjunct tools under mandatory human oversight.

**Supplementary Information:**

The online version contains supplementary material available at https://doi.org/10.1186/s12911-026-03748-y.

## Introduction

The increasing integration of artificial intelligence (AI) in healthcare has transformed medical education and clinical practice. Large Language Models (LLMs), such as ChatGPT, Claude, and Gemini, enable advanced conversational systems that can simulate human-like dialogue and serve as supplementary educational tools [1–3]. However, despite their rising use as medical information sources, significant uncertainty remains regarding their ability to provide guideline-consistent responses. This is particularly critical in high-risk domains such as anesthesia and sedation, where hallucinations or outdated information can pose substantial clinical risks [4, 7].

Pediatric dental procedures frequently require behavioral guidance or advanced behavior management techniques, such as sedation or general anesthesia (GA), especially in very young children or patients with special healthcare needs who are unable to cooperate during treatment [5]. Clinical guidelines issued by major organizations—such as the American Society of Anesthesiologists (ASA), the American Academy of Pediatrics (AAP), the American Dental Association (ADA), and the American Academy of Pediatric Dentistry (AAPD)—provide structured protocols to ensure the safe and effective application of sedation and GA in dental settings [5, 6]. Given the multidisciplinary nature of these interventions, pediatric dentists, anesthesiologists, and oral and maxillofacial surgeons must collaboratively assess patients, determine risk factors, and implement safety protocols [5, 6].

The accurate dissemination of up-to-date information on sedation and general anesthesia is critical for maintaining patient safety. While previous studies have evaluated chatbot performance in general medicine, there is a distinct lack of comparative evidence regarding their reliability in the specialized intersection of pediatric dentistry and anesthesia. The extent to which AI outputs align with the combined expertise of multidisciplinary specialists in this high-stakes field remains unexplored. This study addresses this knowledge gap by evaluating the adherence of multiple AI models to established safety guidelines (such as ASA and AAPD) and benchmarking their accuracy against the clinical profiles of pediatric dentists, anesthesiologists, and oral and maxillofacial surgeons [1, 3, 7].

This study aims to evaluate the accuracy of three advanced chatbot models (ChatGPT 4.0 Mini, Gemini 1.5 Pro, and Claude 3 Sonnet) in providing correct information about pediatric sedation and GA, and to compare their performance with that of specialists from three clinical fields: anesthesiology, pediatric dentistry, and oral and maxillofacial surgery. We hypothesized that while LLMs would demonstrate high theoretical accuracy due to their vast training data, human specialists (particularly anesthesiologists) would show superior performance in categories requiring integrated clinical judgment and patient-specific evaluation [7, 8].

## Methods

### Study design and ethical approval

This study was designed as a descriptive and comparative cross-sectional study to assess the information accuracy of large language models (LLMs) and compare their performance with that of human experts in the context of pediatric sedation and general anesthesia. The study protocol was approved by the Biruni University Ethics Committee for Scientific Research. (Protocol No:2024-BİAEK/11–30 Date: 30.06.2025). Informed consent was obtained from all human participants electronically prior to their participation.

### Question form development and validation

A 26-item true/false questionnaire was developed based on up-to-date clinical guidelines from the ASA, AAPD, and ADA [5, 6]. The items in the questionnaire were derived directly from the source guidelines. For the ‘True’ options, the original sentences from the official guidelines were utilized verbatim or paraphrased without altering their core meaning. Conversely, the ‘False’ options were generated by purposefully altering specific words, operational terms, or numerical values within those same guideline sentences. A preliminary pool of clinical questions was developed by the authors, strictly based on the current clinical practice guidelines. The focus was maintained on critical core competencies. To ensure a comprehensive evaluation, the questions were categorized into six clinical domains: (1) General Information on Sedation and GA, (2) Patient Evaluation and Preparation, (3) Risk Factors and Complications, (4) Special Considerations and High-Risk Groups, and (5) Drugs Used and Their Effects, (6) Postoperative Monitoring and Discharge Criteria.

To ensure content validity and clinical relevance, the initial questionnaire was developed by pediatric dentistry researchers and subsequently reviewed by an expert panel consisting of one anesthesiologist, one pediatric dentist and one oral and maxillofacial surgeon.

### Participant recruitment

A formal power analysis was conducted using G*Power 3.1.9.2 to justify the sample size and ensure maximum statistical power across a 6-arm comparative design (comprising three human specialist —pediatric dentists, oral and maxillofacial surgeons, and anesthesiologists— and three AI chatbot models). Assuming a medium effect size of 0.30, an alpha level of 0.05, and a target power of 0.80, the minimum required total sample size was calculated as 143, dictating approximately 24 independent observations per evaluation group to perform a balanced non-parametric analysis. To maintain strict analytical symmetry and equivalent statistical weight across all arms, a sample size of 24 was assigned to each of the six groups. For the human cohort, this number represented 24 distinct clinical specialists recruited per discipline through a purposive sampling method. For the AI cohort, to dynamically capture the inherent stochastic variations of large language models while establishing a mathematically balanced dataset, the 24 observations were generated by executing 24 independent, distinct inquiry sessions per chatbot model, conducted at different time intervals sessions under cleared-cache conditions. This structural approach successfully avoided an unbalanced design and provided a total matrix of 144 standardized data points (72 from human expert clinical profiles and 72 from AI-generated simulation outputs) optimized for robust statistical comparisons.

The survey form was distributed to 100 participants based on the inclusion criteria per each group through the mailing lists of professional associations. A purposive sampling method was used and data collection was closed once each subgroup reached 24 respondents. The overall response rate was approximately 24% (72 out of 300 invitees).

Consequently, the AI outputs were analyzed not at a ‘participant’ level, but at the level of standardized question-response outputs. The objective of this approach was not to equate AI models with individual human participants, but to examine the accuracy of their generated responses within a comparative evaluation framework alongside human specialist groups. Just as responses to different questions represent diverse components of knowledge in human groups, each question-response output in AI models represents a distinct aspect of model performance.

### Chatbot evaluation and prompting strategy

The questionnaire was administered to three state-of-the-art LLMs: ChatGPT 4.0 Mini (OpenAI), Gemini 1.5 Pro (Google), and Claude 3 Sonnet (Anthropic). Each of the 26 questions was directed to the chatbots between October 6, 2025, and January 9, 2026, at various random and different time intervals after clearing browser cache. To ensure independence, the same question was never asked consecutively, and a new chat session was initiated for each query. To account for the inherent stochastic nature of large language models, where responses may exhibit probabilistic variations, a single-shot query approach was avoided. Instead, as a methodological precaution and to maintain a balanced comparison with the sample size of the human specialist groups (n = 24), the entire questionnaire was administered to each AI model across 24 separate, independent iterations conducted at different time intervals. This repetitive prompting protocol was utilized to stabilize the models’ baseline outputs, evaluate internal response consistency, and ensure that the final data represented an accurate consensus performance rather than an isolated, random variation. A standardized zero-shot prompting technique -where models were queried directly without providing prior examples or contextual training prompts- was utilized to ensure consistency across models. Each chatbot was provided with the following uniform command: ‘Is the following statement true or false?’ immediately followed by the survey item. Only the initial response (True/False) was recorded for analysis. Two independent pediatric dentistry specialists, blinded to the specific model’s identity during the initial coding phase, evaluated each response. Discrepancies were resolved through consensus. All three chatbot models provided explicit ‘True’ or ‘False’ responses for every survey item, allowing for a definitive binary coding process. No neutral, ambiguous, or non-committal answers (such as ‘It depends’ or ‘I am not sure’) were generated during the sessions. This was ensured by the specific prompting instruction which requested a ‘direct answer.’ Consequently, 100% of the chatbot outputs were eligible for analysis, eliminating the need for a ‘neutral’ or ‘excluded’ category in the evaluation phase.

### Statistical analysis

The assumption of normal distribution was verified using the Shapiro-Wilk test. For the comparison of three or more independent groups that did not follow a normal distribution, the Kruskal-Wallis test was employed. To identify the specific group or groups driving the significant difference, Post Hoc Adjusted Bonferroni tests were performed. For testing the relationship between categorical variables, the Pearson Chi-Square test was applied in cases where the sample size assumption (expected value > 5) was met. All statistical analyses were conducted using the IBM Statistical Package for Social Sciences (SPSS), Windows version 27 (SPSS Inc., Chicago, IL, USA).

## Results

### Overall accuracy comparison

**Fig. 1:**
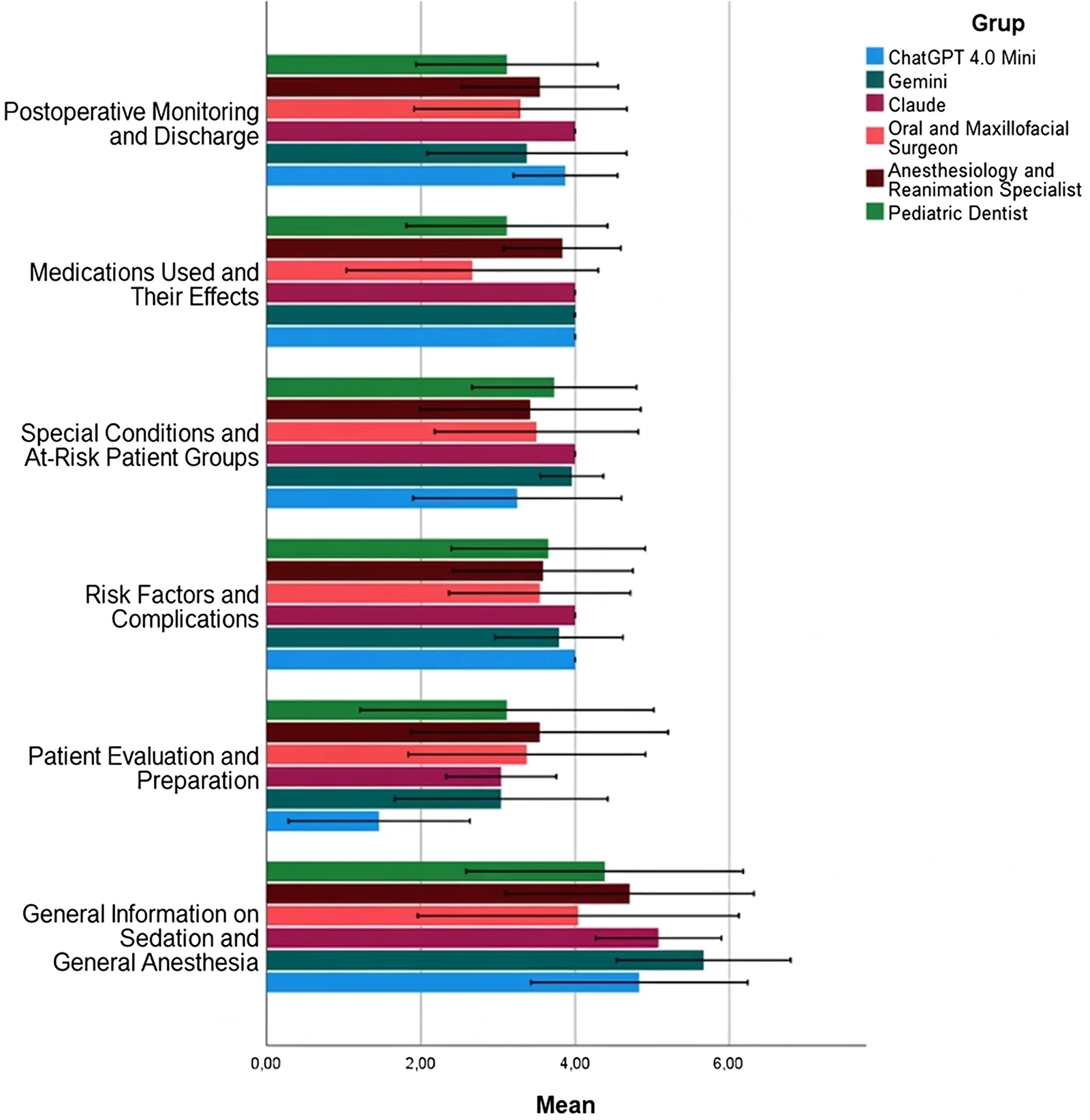
Bar chart illustrating the distribution of knowledge scores across study groups by question type

Statistical analysis revealed a significant difference in the overall accuracy scores among the six study groups (*p* < 0.001, Tables 1 and 2; Fig. 1). Among the Large Language Models (LLMs), Claude 3 Sonnet demonstrated the highest overall accuracy (92.8%), followed by Gemini 1.5 Pro (91.7%) and ChatGPT 4.0 Mini (82.4%). Within the human expert groups, anesthesiology specialists outperformed their peers with an average accuracy of 87.0%. Pediatric dentistry specialists and oral and maxillofacial surgeons (OMFS) achieved accuracy rates of 81.2% and 78.5%, respectively. Notably, the top-performing AI models (Claude 3 Sonnet and Gemini 1.5 Pro) fell into a higher percentage range than those recorded by the human specialist groups.

**Table 1:** Distribution and comparison of knowledge scores by study groups

Clinical domains	Chatgpt 4.0 mini (mean ± sd)	Gemini 1.5 pro (mean ± sd)	Claude 3 sonnet (mean ± sd)	Oral & maxillofacial surgeons (mean ± sd)	Anesthesiology specialists (mean ± sd)	Pediatric dentistry specialists (mean ± sd)	Test stat. (h)	*p*
General ınfo on sedation & ga	4.83 ± 0.70	5.67 ± 0.56	5.08 ± 0.41	4.04 ± 1.04	4.71 ± 0.81	4.38 ± 0.90	47.079	< 0.001*
Patient evaluation & prep	1.46 ± 0.59	3.04 ± 0.69	3.04 ± 0.36	3.38 ± 0.77	3.54 ± 0.83	3.12 ± 0.95	60.515	< 0.001*
Risk factors & complications	4.00 ± 0.00	3.79 ± 0.41	4.00 ± 0.00	3.54 ± 0.59	3.58 ± 0.58	3.65 ± 0.63	22.894	< 0.001*
Special cons. & high-risk groups	3.25 ± 0.68	3.96 ± 0.20	4.00 ± 0.00	3.50 ± 0.66	3.42 ± 0.72	3.73 ± 0.53	34.767	< 0.001*
Drugs used and their effects	4.00 ± 0.00	4.00 ± 0.00	4.00 ± 0.00	2.67 ± 0.82	3.83 ± 0.38	3.12 ± 0.65	86.617	< 0.001*
Postoperative monitoring & discharge	3.88 ± 0.34	3.38 ± 0.65	4.00 ± 0.00	3.29 ± 0.69	3.54 ± 0.51	3.12 ± 0.59	43.036	< 0.001*
Total score	21.42 ± 1.02	23.83 ± 1.01	24.13 ± 0.54	20.42 ± 2.69	22.63 ± 1.81	21.12 ± 2.14	66.472	< 0.001*

*p < 0.05 (Kruskal-Wallis Test). SD: Standard Deviation. Total score is based on a maximum of 24 points

**Table 2:** Comparison of correct response rates between groups

Knowledge domains	Study groups	Incorrect *n* (%)	Correct *n* (%)	Test stat.	*P*-value	Effect size
General ınformation on sedation and general anesthesia	ChatGPT 4.0 Mini	28 (19.4%)	116 (80.6%)	39.545	< 0.001*	0.212
	Gemini	8 (5.6%)	136 (94.4%)			
	Claude	22 (15.3%)	122 (84.7%)			
	Oral & Maxillofacial Surgeon	47 (32.6%)	97 (67.4%)			
	Anesthesiology Specialist	31 (21.5%)	113 (78.5%)			
	Pediatric Dentistry Specialist	42 (26.9%)	114 (73.1%)			
Patient assessment and preparation	ChatGPT 4.0 Mini	61 (63.5%)	35 (36.5%)	85.819	< 0.001*	0.383
	Gemini	23 (24.0%)	73 (76.0%)			
	Claude	23 (24.0%)	73 (76.0%)			
	Oral & Maxillofacial Surgeon	15 (15.6%)	81 (84.4%)			
	Anesthesiology Specialist	11 (11.5%)	85 (88.5%)			
	Pediatric Dentistry Specialist	23 (22.1%)	81 (77.9%)			
Risk factors and complications	ChatGPT 4.0 Mini	0 (0.0%)	96 (100.0%)	22.076	< 0.001*	0.194
	Gemini	5 (5.2%)	91 (94.8%)			
	Claude	0 (0.0%)	96 (100.0%)			
	Oral & Maxillofacial Surgeon	11 (11.5%)	85 (88.5%)			
	Anesthesiology Specialist	10 (10.4%)	86 (89.6%)			
	Pediatric Dentistry Specialist	9 (8.7%)	95 (91.3%)			
Special conditions and risky groups	ChatGPT 4.0 Mini	18 (18.8%)	78 (81.3%)	34.127	< 0.001*	0.242
	Gemini	1 (1.0%)	95 (99.0%)			
	Claude	0 (0.0%)	96 (100.0%)			
	Oral & Maxillofacial Surgeon	12 (12.5%)	84 (87.5%)			
	Anesthesiology Specialist	14 (14.6%)	82 (85.4%)			
	Pediatric Dentistry Specialist	7 (6.7%)	97 (93.3%)			
Drugs used and their effects	ChatGPT 4.0 Mini	0 (0.0%)	96 (100.0%)	109.658	< 0.001*	0.433
	Gemini	0 (0.0%)	96 (100.0%)			
	Claude	0 (0.0%)	96 (100.0%)			
	Oral & Maxillofacial Surgeon	32 (33.3%)	64 (66.7%)			
	Anesthesiology Specialist	4 (4.2%)	92 (95.8%)			
	Pediatric Dentistry Specialist	23 (22.1%)	81 (77.9%)			
Postoperative monitoring & discharge	ChatGPT 4.0 Mini	3 (3.1%)	93 (96.9%)	34.960	< 0.001*	0.245
	Gemini	15 (15.6%)	81 (84.4%)			
	Claude	0 (0.0%)	96 (100.0%)			
	Oral & Maxillofacial Surgeon	17 (17.7%)	79 (82.3%)			
	Anesthesiology Specialist	11 (11.5%)	85 (88.5%)			
	Pediatric Dentistry Specialist	23 (22.1%)	81 (77.9%)			
Total score	ChatGPT 4.0 Mini	110 (17.6%)	514 (82.4%)	86.650	< 0.001*	0.151
	Gemini	52 (8.3%)	572 (91.7%)			
	Claude	45 (7.2%)	579 (92.8%)			
	Oral & Maxillofacial Surgeon	134 (21.5%)	490 (78.5%)			
	Anesthesiology Specialist	81 (13.0%)	543 (87.0%)			
	Pediatric Dentistry Specialist	127 (18.8%)	549 (81.2%)			

*p < 0.05; %: Row percentage; %C: Column percentage. Lowercase letters indicate significant differences between column proportions within a row, while uppercase letters indicate significant differences between row proportions within a column

### Performance by clinical topic category

The performance of both AI models and human experts varied significantly across the six clinical domains (*p* < 0.001, Tables 1 and 2):

- **General Information on Sedation and GA**: Gemini 1.5 Pro achieved the highest accuracy (94.4%), followed by Claude 3 Sonnet (84.7%) and anesthesiology specialists (78.5%).
- **Patient Evaluation and Preparation**: In the ‘Patient Evaluation and Preparation’ category, professional specialist groups generally achieved higher accuracy rates than the LLMs. While anesthesiology specialists and OMFS showed the highest proficiency (88.5% and 84.4%, respectively), Gemini 1.5 Pro and Claude 3 Sonnet (both 76.0%) performed comparably to pediatric dentistry specialists (77.9%). ChatGPT 4.0 Mini was the only model that showed marked numerical lower performance in this domain with 36.5% accuracy.
- **Risk Factors and Complications**: ChatGPT 4.0 Mini and Claude 3 Sonnet achieved 100.0% accuracy, while pediatric dentistry specialists (91.3%) led the human groups.
- **Special Conditions and High-Risk Groups**: Claude 3 Sonnet (100.0%) and Gemini 1.5 Pro (99.0%) showed near-perfect scores, with pediatric dentistry specialists (93.3%) demonstrating the highest proficiency among clinical experts.
- **Drugs Used and Their Effects**: All three AI models (ChatGPT 4.0 Mini, Gemini 1.5 Pro, and Claude 3 Sonnet) achieved 100% accuracy in this theoretical domain. In contrast, OMFS group had the lowest accuracy rate (66.7%) for pharmacological queries.
- **Postoperative Monitoring and Discharge**: Claude 3 Sonnet reached 100% accuracy, while Gemini (84.4%) and OMFS (82.3%) demonstrated relatively lower performance compared to other categories.

### Inter-group and Inter-topic comparisons

Analysis of accuracy rates across different categories revealed distinct performance patterns among the study groups. The Large Language Models (LLMs)—ChatGPT 4.0 Mini and Claude 3 Sonnet —demonstrated maxiumum possible scores ‘Risk Factors and Complications’ and ‘Drugs Used and Their Effects’ with 100.0% accuracy; Gemini 1.5 Pro followed closely, achieving 100.0% in drugs but 94.8% in risk factors. However, a notable reduction in score was observed in the “Patient Assessment and Preparation” category for all models, with ChatGPT 4.0 Mini recording the lowest accuracy at 36.5%.

Professional groups showed varying areas of expertise. Oral and Maxillofacial Surgeons achieved their highest scores in “Risk Factors and Complications” (88.5%) and " Special Considerations and High-Risk Groups” (87.5%), while showing lower accuracy in “Drugs Used and Their Effects” (66.7%). Similarly, Anesthesiology and Reanimation Specialists peaked in the “Drugs Used and Their Effects” category (95.8%), with lower relative performance in “General Information on Sedation and General Anesthesia” (78.5%).

Pediatric Dentistry Specialists exhibited high accuracy in " Special Considerations and High-Risk Groups” (93.3%) and “Risk Factors and Complications” (91.3%). Consistent with other professional groups, their lowest accuracy rate was observed in the “General Information on Sedation and General Anesthesia” category (73.1%). Overall, while LLMs demonstrated high proficiency in extracting pharmacological and risk-related theoretical knowledge, professional specialists maintained more consistent, though numerically lower, scores across clinical preparation and assessment categories compared to the AI models.

Post-hoc comparisons using Bonferroni-adjusted p-values confirmed statistically significant differences across categories within each group (*p* < 0.001). Specifically, for ChatGPT 4.0 Mini, performance in “Patient Evaluation” was significantly lower than in all other categories. Among human participants, anesthesiologists showed more consistent performance across domains compared to OMFS, whose performance dropped significantly in the “Drugs Used” category.

## Discussion

This study evaluated the accuracy of three widely used AI chatbots—ChatGPT 4.0 Mini, Gemini 1.5 Pro, and Claude 3 Sonnet—on pediatric sedation and general anesthesia topics by comparing their performance against expert clinicians. Our findings demonstrate that Claude 3 Sonnet (92.8%) and Gemini 1.5 Pro (91.7%) achieved exceptionally high accuracy in retrieving textbook information, falling slightly above all human expert groups in the overall test scores without implying clinical superiority.

To bridge the gap between theoretical knowledge and real-world patient safety, the clinical significance of these accuracy rates must be translated into potential clinical error rates. For instance, while a 91.7% accuracy rate appears high under test conditions, the reciprocal 8.3% error rate carries critical implications in active clinical practice. Extrapolated across a hypothetical cohort of 1,000 pediatric sedation or general anesthesia cases, an 8.3% error rate mathematically translates into approximately 83 instances where a clinical decision could be compromised by inaccurate or flawed information.

### Analysis of AI and human expert scores

The prominent overall accuracy scores achieved by Claude 3 Sonnet and Gemini 1.5 Pro aligns with a growing body of literature suggesting that large language models (LLMs) are becoming increasingly proficient in handling theoretical medical and dental knowledge domains [1, 2]. Recent evidence highlights that LLMs can be successfully calibrated with human subject matter experts to grade clinical responses in dental education, often matching or exceeding human performance in theoretical knowledge assessment [26]. Furthermore, our methodological framework, which benchmarks AI accuracy against clinical guidelines (ASA, AAPD, ADA, AAP), is consistent with other comparative studies in the field. For instance, similar benchmarking approaches have demonstrated the utility of LLMs in verifying complex surgical protocols, such as intentional replantation [29], as well as achieving diagnostic sensitivity comparable to experienced dental specialists in identifying periapical pathosis [27].

These results reinforce the trajectory of AI as a viable informational reference tools or supplementary educational resources provided it undergoes further clinical validation [3]. In high-stakes scenarios, such as managing pediatric laryngospasm, AI chatbots might theoretically serve as ‘just-in-time’ review tools during simulation-based training or post-hoc case analysis to reinforce expert memory [7]. This is particularly relevant since experienced clinicians can experience ‘cognitive tunneling’ during acute crises, which increases cognitive load during real-time decision-making [8, 9]. Recent evaluations of advanced LLMs in complex medical licensing tasks, including the USMLE exams, have further demonstrated their enhanced performance over smaller, specialized models [10–12]. In contrast, ChatGPT 4.0 Mini achieved an accuracy of 82.4% in our study, falling below the other LLMs and anesthesiology experts (87.0%). This discrepancy suggests that model architecture and training data scope significantly influence performance, echoing previous findings where Claude outperformed older or scaled-down GPT models in dental diagnostic accuracy [13].

### Topic-specific variability: human intuition vs. AI data retrieval

A pivotal finding was the discrepancy in the ‘Patient Evaluation and Preparation’ domain, where anesthesiology specialists (88.5%) and oral surgeons (84.4%) outperformed all AI models. This observation aligns with recent research indicating that while LLMs excel at information retrieval, their diagnostic reasoning varies significantly across domains and requires strict integration with clinical findings [30]. While Claude 3 Sonnet and Gemini 1.5 Pro showed high theoretical knowledge, their lower scores in pre-operative assessment (76.0% for both) highlight a distinct limitation in processing the nuanced clinical reasoning required for patient preparation. This gap underscores that while AI can efficiently process vast medical literature, it struggles with the complex, multi-factorial synthesis of patient data that characterizes human judgment [14].

Conversely, the performance of oral and maxillofacial surgeons (OMFS) in sedation pharmacology—where an overall accuracy of 66.7% was recorded—variability in recalling detailed pharmacological guidelines [15]. This finding reinforces the notion that professional expertise and AI capabilities are complementary. Anesthesiologists provide the necessary clinical safety net, while AI can compensate for human variability in rapidly evolving pharmacological guidelines where surgical specialists may face recall changelles due to distinct clinical priorities. The 66.7% accuracy in the OMFS group may stem from a small sample size, lower clinical exposure to pediatric patients compared to pedodontists and anesthesiologists, and a residency curriculum that prioritizes advanced surgery over deep pediatric pharmacology. Rather than an educational failure, this reflects a practical focus divergence. Consequently, this domain represents an ideal opportunity to integrate AI into the clinical workflow. By using LLMs as real-time cognitive aids at the point of care, OMFS specialists can rapidly verify pediatric dosages and drug interactions, effectively bridging curriculum-driven knowledge boundaries. Consequently, the future of pediatric sedation will likely evolve toward a synergistic ‘centaur’ model, where AI’s systematic data processing complements human clinical judgment [16]. Similar patterns of AI acting as an effective clinical adjunct have been observed in other pediatric emergency scenarios [17], orthodontics [18], and dental trauma [19], particularly when benchmarking adherence to specific clinical guidelines like those of the AAPD [20, 21].

### Implications for education and model integrity

The integration of AI into dental education remains promising for knowledge reinforcement. Our data showed that chatbots consistently provided correct answers in ‘Postoperative Monitoring and Discharge’, suggesting their potential as supplementary educational resources under specialist supervision. Previous studies have similarly noted the utility of these models in periodontal and clinical learning [1, 22]. However, critical risks such as “hallucinations” or an inability to interpret patient-specific nuances warrant caution, as they could compromise safety without expert oversight [4].

As AI systems evolve through reinforcement learning, their clinical accuracy will potentially increase [2]. However, real-world implementation demands strict interdisciplinary validation, expert auditing, and addressing the ‘black box’ nature of AI decision-making to ensure transparency and prevent the adoption of biases from the training corpus [23–27].

Beyond statistical metrics, the clinical meaningfulness of these findings must be considered. In pediatric sedation, where the margin for error is narrow, the performance variance between models—such as Gemini’s 91.7% versus ChatGPT 4.0 Mini’s 82.4% overall accuracy and its sharp drop to 36.5% in consistency—is not merely a statistical difference. It represents a tangible risk of informational errors during emergency decision support. Furthermore, a recent systematic review emphasizes that while multimodal and advanced language models offer significant clinical utility, their performance must be rigorously validated across diverse clinical scenarios to guarantee diagnostic reliability and patient safety [28]. Because models like Claude 3 Sonnet displayed lower performance in ‘Patient Evaluation’ compared to anesthesiologists, it is clear that AI still lacks the clinical wisdom necessary for bedside decision-making. Therefore, AI integration into clinical workflows must be measured by its direct impact on patient safety and procedural outcomes rather than standalone theoretical scores.

However, the remarkably high accuracy rates achieved by ChatGPT 4.0 Mini, Gemini 1.5 Pro, and Claude 3 Sonnet in this study must be interpreted with professional caution and should not be automatically equated with human-like clinical reasoning, critical thinking, or genuine diagnostic acumen. Recent landmark studies across various dental and medical disciplines persistently emphasize that LLM performance on standardized text-based assessments often creates an illusion of competence. For instance, in pediatric sedation and dental education, while models demonstrate satisfactory retrieval of theoretical guidelines, they frequently fail to synthesize complex, dynamic clinical cases that demand multi-step human intuition [29, 30]. This distinction underscores that high factual accuracy on structured questionnaires does not guarantee safe or flexible reasoning in a real-world clinical setting.

Furthermore, a critical confounding factor that complicates the evaluation of LLMs in specialized domains like pediatric dentistry, oral medicine, anatomy, and maxillofacial radiology is the phenomenon of data contamination [29, 31]. Data contamination occurs when the testing material—or very similar variants of these clinical questions—has already been included in the massive, undisclosed web-scale datasets used to pre-train these models. Consequently, when an LLM correctly answers a clinical query, it may not be ‘solving’ the medical problem through algorithmic logic; rather, it is merely performing probabilistic pattern-matching or direct memory retrieval. Recent literature evaluating LLM performance in specialized oral medicine and maxillofacial radiology confirms that models perform exceptionally well on historical or deeply entrenched textbook paradigms, yet their performance drops significantly when faced with novel, image-dependent, or un-contaminated prompts [31, 32]. Therefore, while the high scores observed in our study confirm the robust data-retrieval capabilities of contemporary LLMs, they also highlight a crucial baseline limitation: high accuracy may mask a sophisticated memorization mechanism rather than reflecting true critical thinking.

## Limitations

This study is subject to several limitations. Although the questions were strictly developed based on ASA and AAPD guidelines, the true/false format may not fully capture the multi-dimensional complexity and nuanced decision-making of real-world clinical scenarios. Furthermore, while the sample size of human experts provided a robust baseline for comparison, it may not represent the full spectrum of global clinical expertise across different healthcare systems. Furthermore, although the 26-item questionnaire was grounded in international clinical guidelines and expert consensus, it did not undergo prior pilot testing or a formal reliability evaluation. Future studies employing standardized and psychometrically validated instruments could provide more robust comparisons between AI models and human specialists.

Chatbot responses were evaluated using standardized zero-shot prompting under ideal conditions; in practice, the variability of user-generated prompts (prompt engineering) might influence the models’ reliability and output consistency. Since AI models undergo frequent updates and iterative training, the performance recorded in this study reflects a specific temporal snapshot, and newer versions of these models might yield different results. To account for the inherent stochastic nature of generative AI models, this study avoided a single-shot query approach and instead employed a repetitive zero-shot prompting protocol across 24 separate sessions. However, while this repetitive multi-session sampling captured output consistency, it remains bounded by the specific temporal versions of the tested models.

While accuracy was measured quantitatively, qualitative aspects such as the logical structure of clinical reasoning, the depth of explanations, and ethical sensitivity were not assessed.

Only English-language models were evaluated, which may limit the generalizability of these findings to non-English-speaking clinical environments where local guidelines and linguistic nuances might differ.

The baseline demographic characteristics of the expert participants, such as age and years of professional experience, were not recorded. This lack of demographic data limits our ability to fully evaluate whether the groups were perfectly matched, which should be considered when interpreting the inter-group comparisons.

A fundamental methodological constraint of this study is the environmental asymmetry between the human and AI cohorts during evaluation. The human expert groups completed the questionnaire in an unsupervised environment with no strict time limits, allowing potential access to external reference materials—a design choice intended to mirror real-world clinical decision-making and standard consultation habits. Conversely, the AI models were evaluated under rigid, controlled session parameters representing a structured zero-shot data retrieval test without active web-browsing capabilities. This procedural difference in testing environments should be carefully considered when evaluating the comparative performance outcomes.

Finally, the relatively restricted number of human expert participants (*n* = 24 per specialty, total human *N* = 72) constitutes a limitation. While this human sample size was methodologically balanced by integrating a mathematically equivalent number of AI-generated performance outputs to achieve a robust matrix of 144 total observations, a larger human cohort would further strengthen the external validity of the findings. Future global, multi-center studies with a higher number of international participants are warranted to expand the generalizability of these comparisons and provide broader clinical insights.

Further research should explore real-time clinical decision support simulations involving larger, multicenter expert cohorts, assess longitudinal performance across model updates, and integrate qualitative assessments of AI-generated clinical justifications.

## Conclusion

The findings of this study demonstrate that AI-based chatbots, particularly Claude 3 Sonnet and Gemini 1.5 Pro, can achieve competitive accuracy to human specialists within the tested, guideline-based True/False questionnaire in pediatric sedation and anesthesia topics, demonstrating high proficiency in overall theoretical knowledge retrieval. However, the prominent performance of anesthesiologists in ‘Patient Evaluation and Preparation’ underscores that human clinical intuition and the multi-factorial synthesis of patient data remain essential. Furthermore, the significant performance variance observed—specifically the lower numerical consistency of ChatGPT 4.0 Mini in providing guideline-consistent responses—highlights that AI performance is not uniform and remains highly dependent on model architecture.

These results support the integration of advanced LLMs as supplementary informational reference tools and educational aids under specialist supervision. The future of pediatric sedation safety likely resides in a synergistic ‘centaur’ model, where the systematic data-retrieval strengths of AI complement human clinical judgment in navigating rapidly evolving guidelines, while human experts provide the final ethical and clinical safety net. Nevertheless, rigorous interdisciplinary validation and human oversight remain mandatory to mitigate risks such as hallucinations. As AI becomes embedded in healthcare workflows, continuous evaluation of its accuracy and safety is crucial to ensure patient safety in pediatric dentistry.

## Supplementary Information

Below is the link to the electronic supplementary material.

Supplementary Material 1

Supplementary Material 2

Supplementary Material 3

Supplementary Material 4

## Data Availability

The datasets generated and/or analysed during the current study are available from the corresponding author on reasonable request.
