# Supplementary material for: Evaluation of chatbot and specialist knowledge on pediatric sedation and general anesthesia: a comparative analysis
Source: BMC Med Inform Decis Mak. 2026 Aug 5;26:444. doi: 10.1186/s12911-026-03748-y (PMC13725993; doi:10.1186/s12911-026-03748-y)
Supplement: Supplementary file 1 [file 12911_2026_3748_MOESM2_ESM.docx]

**SEDATION AND GENERAL ANESTHESIA QUESTIONS**

**1. General Information on Sedation and General Anesthesia**

1. Patients under minimal sedation can respond normally to verbal commands and light tactile stimulation. (T) **[ASA/AAPD]**
2. A patient who exhibits only a reflex withdrawal response is considered to be under moderate sedation. (F) *(Note: This indicates Deep Sedation)* **[ASA]**
3. Administering a new dose before observing the previous sedation effects can lead to unintended deep sedation. (T) **[AAPD]**
4. Positive pressure ventilation is not required during deep sedation. (F) *(Note: It may be required due to potential respiratory depression)* **[ASA/AAP]**
5. Cardiovascular function is always maintained during general anesthesia. (F) **[ASA]**
6. During general anesthesia, the patient's level of consciousness is completely lost and reflexes are suppressed. (T) **[ASA]**

**2. Patient Evaluation and Preparation**

7. Prior to sedation and general anesthesia, the patient’s ASA physical status classification must be determined, and the airway patency must be assessed during the physical evaluation. (T) **[ASA/AAPD]**
8. Infants breastfed prior to sedation must fast for at least 4 hours. (T) **[ASA/AAP]**
9. Solid foods should be discontinued 2 hours before sedation. (F) *(Note: The requirement is 6 hours for light meals, 8 hours for fatty/fried foods)* **[ASA]**
10. Clear liquids (e.g., water, apple juice) can be consumed up to 30 minutes before sedation. (F) *(Note: The limit is 2 hours)* **[ASA/AAPD]**

**3. Risk Factors and Complications**

11. Airway obstruction is the most common serious complication during sedation. (T) **[AAPD]**
12. The risk of aspiration is low during general anesthesia and does not require precautions. (F) **[ASA]**
13. Children are more sensitive to sedation than adults and have a higher risk of complications. (T) **[AAPD/AAP]**
14. Agents such as halothane and succinylcholine can be safely used in individuals with a genetic predisposition to malignant hyperthermia. (F) **[ASA]**

**4. Special Considerations and High-Risk Patient Groups**

15. In children with developmental disabilities, the risk of desaturation after sedation can increase three-fold. (T) **[AAPD]**
16. The risk of developing apnea is lower in premature infants. (F) *(Note: It is significantly higher)* **[AAP/AAPD]**
17. Distraction techniques can be attempted as an alternative to pharmacological sedation in pediatric patients. (T) **[AAPD]**
18. There is no risk of airway obstruction in patients with mild tonsillar hypertrophy. (F) **[AAPD]**

**5. Drugs Used and Their Effects**

19. The duration of action of Midazolam is longer than that of Diazepam. (F) **[ASA]**
20. Propofol is used as an intravenous induction agent in general anesthesia. (T) **[ASA]**
21. Promethazine is safe for children under 2 years of age. (F) *(Note: It carries a black box warning for respiratory depression in this age group)* **[FDA/AAP]**
22. Sevoflurane is the preferred inhalation anesthetic agent for mask induction in children. (T) **[ASA]**

**6. Postoperative Monitoring and Discharge**

23. Agitation and disorientation are common temporary conditions in pediatric patients after general anesthesia and may require special monitoring. (T) **[AAPD]**
24. The observation period after sedation may vary depending on the drug used. (T) **[ASA/AAPD]**
25. Patients administered reversal agents should be monitored for a shorter period than others. (F) *(Note: They require longer monitoring due to the risk of re-sedation)* **[ASA/AAPD]**
26. A child who can remain awake for 15 minutes after sedation can generally be safely discharged. (F) *(Note: Discharge criteria are more comprehensive, involving cardiovascular stability and protective reflexes)* **[AAPD]**
