# Supplementary material for: Evaluation of chatbot and specialist knowledge on pediatric sedation and general anesthesia: a comparative analysis
Source: BMC Med Inform Decis Mak. 2026 Aug 5;26:444. doi: 10.1186/s12911-026-03748-y (PMC13725993; doi:10.1186/s12911-026-03748-y)
Supplement: Supplementary file 2 [file 12911_2026_3748_MOESM4_ESM.docx]

**Statistical Method**

Descriptive statistics of the data were presented as frequency, percentage, mean, standard deviation, median, minimum, maximum, and interquartile range (25th and 75th percentiles). The normality of the data distribution was evaluated using the Shapiro-Wilk test. For the comparison of three or more independent groups that did not display a normal distribution, the Kruskal-Wallis test was performed. To identify the specific group or groups driving the statistical difference, post hoc Bonferroni-corrected tests were utilized. The relationship between categorical variables was analyzed using the Pearson Chi-Square test, provided that the sample size assumption (expected value >5) was satisfied. All statistical analyses were conducted using the IBM Statistical Package for Social Sciences (SPSS) for Windows, version 27.0 (SPSS Inc., Chicago, IL, USA).

**Tablo…** Çalışma gruplarına göre bilgi puanlarının dağılımları ve karşılaştırılması

| Puanlar | Çalışma Grupları | Min.-Maks. | Ort.±S.S. | M.(%25-%75Ç.) | Test İst. | p | Etki Büy. |
| --- | --- | --- | --- | --- | --- | --- | --- |
| Sedasyon ve Genel Anestezi Genel Bilgiler | ChatGPT 4.0 Mini | 4-6 | 4,83±0,70 | 5(4-5) | 47,0791 | <0,001* | 0,318 |
|  | Gemini | 4-6 | 5,67±0,56 | 6(5-6) |  |  |  |
|  | Claude | 4-6 | 5,08±0,41 | 5(5-5) |  |  |  |
|  | Ağız, Diş ve Çene Cerrahisi Uzmanı | 1-6 | 4,04±1,04 | 4(3,5-5) |  |  |  |
|  | Anestezi ve Reanimasyon Uzmanı | 3-6 | 4,71±0,81 | 5(4-5) |  |  |  |
|  | Çocuk Diş Hekimliği Uzmanı | 3-6 | 4,38±0,90 | 4(4-5) |  |  |  |
| Hasta Değerlendirme ve Hazırlık | ChatGPT 4.0 Mini | 1-3 | 1,46±0,59 | 1(1-2) | 60,515 | <0,001* | 0,476 |
|  | Gemini | 2-4 | 3,04±0,69 | 3(3-3,5) |  |  |  |
|  | Claude | 2-4 | 3,04±0,36 | 3(3-3) |  |  |  |
|  | Ağız, Diş ve Çene Cerrahisi Uzmanı | 2-4 | 3,38±0,77 | 4(3-4) |  |  |  |
|  | Anestezi ve Reanimasyon Uzmanı | 1-4 | 3,54±0,83 | 4(3-4) |  |  |  |
|  | Çocuk Diş Hekimliği Uzmanı | 1-4 | 3,12±0,95 | 3(3-4) |  |  |  |
| Risk Faktörleri ve Komplikasyonlar | ChatGPT 4.0 Mini | 4-4 | 4±0 | 4(4-4) | 22,894 | <0,001* | 0,144 |
|  | Gemini | 3-4 | 3,79±0,41 | 4(4-4) |  |  |  |
|  | Claude | 4-4 | 4±0 | 4(4-4) |  |  |  |
|  | Ağız, Diş ve Çene Cerrahisi Uzmanı | 2-4 | 3,54±0,59 | 4(3-4) |  |  |  |
|  | Anestezi ve Reanimasyon Uzmanı | 2-4 | 3,58±0,58 | 4(3-4) |  |  |  |
|  | Çocuk Diş Hekimliği Uzmanı | 2-4 | 3,65±0,63 | 4(3-4) |  |  |  |
| Özel Durumlar ve Riskli Hasta Grubu | ChatGPT 4.0 Mini | 2-4 | 3,25±0,68 | 3(3-4) | 34,767 | <0,001* | 0,215 |
|  | Gemini | 3-4 | 3,96±0,20 | 4(4-4) |  |  |  |
|  | Claude | 4-4 | 4±0 | 4(4-4) |  |  |  |
|  | Ağız, Diş ve Çene Cerrahisi Uzmanı | 2-4 | 3,50±0,66 | 4(3-4) |  |  |  |
|  | Anestezi ve Reanimasyon Uzmanı | 2-4 | 3,42±0,72 | 4(3-4) |  |  |  |
|  | Çocuk Diş Hekimliği Uzmanı | 2-4 | 3,73±0,53 | 4(4-4) |  |  |  |
| Kullanılan İlaçlar ve Etkileri | ChatGPT 4.0 Mini | 4-4 | 4±0 | 4(4-4) | 86,617 | <0,001* | 0,576 |
|  | Gemini | 4-4 | 4±0 | 4(4-4) |  |  |  |
|  | Claude | 4-4 | 4±0 | 4(4-4) |  |  |  |
|  | Ağız, Diş ve Çene Cerrahisi Uzmanı | 2-4 | 2,67±0,82 | 2(2-3) |  |  |  |
|  | Anestezi ve Reanimasyon Uzmanı | 3-4 | 3,83±0,38 | 4(4-4) |  |  |  |
|  | Çocuk Diş Hekimliği Uzmanı | 2-4 | 3,12±0,65 | 3(3-4) |  |  |  |
| Postoperatif İzlem ve Taburculuk | ChatGPT 4.0 Mini | 3-4 | 3,88±0,34 | 4(4-4) | 43,036 | <0,001* | 0,278 |
|  | Gemini | 2-4 | 3,38±0,65 | 3(3-4) |  |  |  |
|  | Claude | 4-4 | 4±0 | 4(4-4) |  |  |  |
|  | Ağız, Diş ve Çene Cerrahisi Uzmanı | 2-4 | 3,29±0,69 | 3(3-4) |  |  |  |
|  | Anestezi ve Reanimasyon Uzmanı | 3-4 | 3,54±0,51 | 4(3-4) |  |  |  |
|  | Çocuk Diş Hekimliği Uzmanı | 2-4 | 3,12±0,59 | 3(3-3) |  |  |  |
| Toplam | ChatGPT 4.0 Mini | 20-24 | 21,42±1,02 | 21(21-22) | 66,472 | <0,001* | 0,405 |
|  | Gemini | 23-26 | 23,83±1,01 | 23,5(23-24,5) |  |  |  |
|  | Claude | 23-25 | 24,13±0,54 | 24(24-24) |  |  |  |
|  | Ağız, Diş ve Çene Cerrahisi Uzmanı | 15-25 | 20,42±2,69 | 20,5(19,5-22) |  |  |  |
|  | Anestezi ve Reanimasyon Uzmanı | 19-25 | 22,63±1,81 | 22,5(22-24) |  |  |  |
|  | Çocuk Diş Hekimliği Uzmanı | 17-25 | 21,12±2,14 | 21(20-23) |  |  |  |

*p<0,05

Bu yoğun bulgular (Results) metnini, uluslararası yüksek indeksli (Q1/Q2) dergilerin formatına uygun, tekrarlardan arındırılmış, son derece akıcı ve kesin bir akademik İngilizceye çevirdim.Metnin uzun p-değeri listelerini ve "kim kimden yüksek" karşılaştırmalarını hakemlerin ve editörlerin en sevdiği yapı olan "sırasıyla" (respectively) ve "significantly higher" kalıplarıyla birleştirerek rafine hale getirdim.Metinde gözüme çarpan bir iki küçük maddi/yazım hatasını da (örneğin orijinal metindeki "Anestezi ve Reanimasyon Uzmanı Mini" ifadesindeki fazlalık "Mini" kelimesini ve "Ağız, Diş ve Çene Cerrahisi Uzman" kısmındaki eksik takıyı) çeviri esnasında akademik doğruluk adına kendiliğinden düzelttim.İşte makalenize doğrudan ekleyebileceğiniz akademik çeviri:Statistical Results (Bulgular)The distributions of knowledge scores across the study groups are presented in the table and were compared using Kruskal-Wallis tests. The analyses revealed statistically significant differences among the study groups across all knowledge scores ($p < 0.05$).Regarding "General Information on Sedation and General Anesthesia," post hoc Bonferroni-corrected tests indicated significant differences between Gemini and oral and maxillofacial surgeons ($p < 0.001$), pediatric dentists ($p < 0.001$), anesthesiologists ($p = 0.001$), and ChatGPT 4.0 Mini ($p = 0.005$), as well as between Claude and oral and maxillofacial surgeons ($p = 0.003$). Specifically, Gemini's knowledge scores were significantly higher than those of oral and maxillofacial surgeons, pediatric dentists, and anesthesiologists, while Claude's scores were significantly higher than those of oral and maxillofacial surgeons.For "Patient Evaluation and Preparation," Bonferroni-corrected tests revealed significant differences between ChatGPT 4.0 Mini and all other groups, including Claude, Gemini, oral and maxillofacial surgeons, pediatric dentists, and anesthesiologists ($p < 0.001$). The knowledge scores of Claude, Gemini, oral and maxillofacial surgeons, pediatric dentists, and anesthesiologists were significantly higher than those of ChatGPT 4.0 Mini.In terms of "Risk Factors and Complications," significant differences were observed between ChatGPT 4.0 Mini and Claude versus oral and maxillofacial surgeons and anesthesiologists ($p = 0.007$, $p = 0.025$, $p = 0.007$, and $p = 0.025$, respectively). The knowledge scores of ChatGPT 4.0 Mini and Claude were significantly higher than those of oral and maxillofacial surgeons and anesthesiologists.For "Special Conditions and High-Risk Patient Groups," significant differences were detected between ChatGPT 4.0 Mini and pediatric dentists, Gemini, and Claude; between anesthesiologists and Gemini and Claude; and between oral and maxillofacial surgeons and Claude ($p = 0.037$, $p < 0.001$, $p < 0.001$, $p = 0.020$, $p = 0.007$, and $p = 0.028$, respectively). The knowledge scores of pediatric dentists, Gemini, and Claude were significantly higher than those of ChatGPT 4.0 Mini. Furthermore, Gemini and Claude scored significantly higher than anesthesiologists, while Claude scored significantly higher than oral and maxillofacial surgeons.Regarding "Medications and Their Effects," Bonferroni-corrected tests demonstrated significant differences between oral and maxillofacial surgeons and pediatric dentists versus anesthesiologists, ChatGPT 4.0 Mini, Gemini, and Claude ($p < 0.001$). The knowledge scores of anesthesiologists, ChatGPT 4.0 Mini, Gemini, and Claude were significantly higher than those of oral and maxillofacial surgeons and pediatric dentists.For "Postoperative Monitoring and Discharge," significant differences were found between ChatGPT 4.0 Mini and Claude versus pediatric dentists, oral and maxillofacial surgeons, and Gemini, as well as between Claude and anesthesiologists ($p < 0.001$, $p = 0.013$, $p = 0.048$, $p < 0.001$, $p < 0.001$, $p = 0.003$, and $p = 0.043$, respectively). The knowledge scores of ChatGPT 4.0 Mini and Claude were significantly higher than those of pediatric dentists, oral and maxillofacial surgeons, and Gemini. Additionally, Claude's scores were significantly higher than those of anesthesiologists.Finally, for "Total Scores," Bonferroni-corrected tests revealed significant differences between Gemini and Claude versus oral and maxillofacial surgeons, ChatGPT 4.0 Mini, and pediatric dentists, as well as between anesthesiologists versus Claude and oral and maxillofacial surgeons ($p < 0.001$ for the first six comparisons; $p = 0.048$ and $p = 0.037$, respectively). The total knowledge scores of Gemini and Claude were significantly higher than those of oral and maxillofacial surgeons, ChatGPT 4.0 Mini, and pediatric dentists. Claude scored significantly higher than anesthesiologists, and anesthesiologists scored significantly higher than oral and maxillofacial surgeons.

Bu bulgular (Results) metni, Pearson Ki-Kare testi sonuçlarını ve kategorik veri yüzdelerini içeriyor. Metni, uluslararası tıp ve diş hekimliği dergilerinin (özellikle anestezi ve yapay zeka odaklı yüksek indeksli yayınların) standartlarına uygun, tekrarlardan kaçınan, akıcı ve net bir akademik İngilizceye çevirdim.Metindeki oransal karşılaştırmaları daha profesyonel hale getirmek adına "the highest", "the lowest", "comparable to" (birbirine benzer) ve "significantly higher/lower" gibi tam akademik karşılıkları kullandım. Orijinal metinde yer almayan ancak "Kullanılan İlaçlar ve Etkileri" başlığının bu paragrafta eksik olduğunu fark ettim; metninizde ne yazıyorsa sadık kalarak eksiksiz bir şekilde aktardım.İşte makaleniz için hazırlanan akademik çeviri:Statistical Results: Categorical Data Analysis (Bulgular)The distribution of responses according to the study groups is presented in the table, and Pearson Chi-Square tests were performed to analyze the relationships between them. The analyses revealed statistically significant relationships between the study groups and the responses across all headings ($p < 0.05$).Regarding "General Information on Sedation and General Anesthesia," the correct response rate for ChatGPT 4.0 Mini was 80.6%. In the Gemini group, this rate was 94.4%, which was statistically significantly higher than all other groups. The correct response rate in the Claude group was 84.7%; while this was lower than Gemini, it was statistically significantly higher than that of oral and maxillofacial surgeons (67.4%). The oral and maxillofacial surgery group demonstrated the lowest correct response rate at 67.4%. The correct response rates for anesthesiologists and pediatric dentists were 78.5% and 73.1%, respectively, both being notably lower than the Gemini group.For "Patient Evaluation and Preparation," the ChatGPT 4.0 Mini group exhibited the lowest correct response rate at 36.5%. The correct response rates for the Gemini and Claude groups were both 76.0%, which were statistically significantly higher than that of the ChatGPT 4.0 Mini group. Anesthesiologists achieved the highest correct response rate at 88.5%, closely followed by oral and maxillofacial surgeons at 84.4%. Pediatric dentists demonstrated a correct response rate of 77.9%, which was also significantly higher than that of ChatGPT 4.0 Mini.In terms of "Risk Factors and Complications," ChatGPT 4.0 Mini and Claude groups achieved a 100.0% correct response rate, representing the highest performance. The correct response rate for Gemini was 94.8%; while this was lower than ChatGPT 4.0 Mini and Claude, it was notably higher than those of oral and maxillofacial surgeons (88.5%), anesthesiologists (89.6%), and pediatric dentists (91.3%). The correct response rates among oral and maxillofacial surgeons, anesthesiologists, and pediatric dentists were comparable to one another and statistically significantly lower than those of the ChatGPT 4.0 Mini and Claude groups.For "Special Conditions and High-Risk Patient Groups," the Claude group achieved the highest correct response rate at 100.0%. The Gemini group followed closely with a 99.0% correct response rate, demonstrating high performance and scoring statistically significantly higher than ChatGPT 4.0 Mini (81.3%), oral and maxillofacial surgeons (87.5%), and anesthesiologists (85.4%). The correct response rates for ChatGPT 4.0 Mini, oral and maxillofacial surgeons, and anesthesiologists were comparable, and these groups scored significantly lower than Gemini and Claude. Pediatric dentists achieved a correct response rate of 93.3%, which was higher than ChatGPT 4.0 Mini but lower than Gemini and Claude.Regarding "Postoperative Monitoring and Discharge," the Claude group showed the highest performance with a 100.0% correct response rate. The correct response rate for ChatGPT 4.0 Mini was 96.9%, which was high and comparable to Claude. Anesthesiologists achieved a 88.5% correct response rate, which was notably higher than the rates of Gemini (84.4%), oral and maxillofacial surgeons (82.3%), and pediatric dentists (77.9%). The correct response rates for Gemini, oral and maxillofacial surgeons, and pediatric dentists were comparable and relatively lower.Finally, when the "Total Correct Response Rates" were analyzed, the Claude group achieved the highest overall performance at 92.8%. The Gemini group followed closely with a 91.7% correct response rate, indicating a similarly high level of accuracy. Anesthesiologists demonstrated a 87.0% correct response rate, performing significantly better than ChatGPT 4.0 Mini (82.4%) and pediatric dentists (81.2%). Oral and maxillofacial surgeons exhibited the lowest total correct response rate at 78.5%, which was statistically significantly lower than all other study groups.

The distribution of responses by topics for each study group is presented in the table, and Pearson Chi-Square tests were performed to evaluate the relationships between them. The analyses demonstrated statistically significant relationships between the topics and response categories within all study groups ($p < 0.05$).In the ChatGPT 4.0 Mini group, the correct response rate reached its highest level at 100.0% in both "Risk Factors and Complications" and "Medications and Their Effects." The correct response rate in "Postoperative Monitoring and Discharge" (96.9%) was significantly higher than those in "General Information on Sedation and General Anesthesia" (80.6%) and "Special Conditions and High-Risk Patient Groups" (81.3%). Conversely, the correct response rate in "Patient Evaluation and Preparation" was the lowest among all topics at 36.5%.In the Gemini group, the highest correct response rate was observed in "Medications and Their Effects" at 100.0%. High and comparable rates were obtained in "Special Conditions and High-Risk Patient Groups" (99.0%), "General Information on Sedation and General Anesthesia" (94.4%), and "Risk Factors and Complications" (94.8%). The correct response rates in "Postoperative Monitoring and Discharge" (84.4%) and "Patient Evaluation and Preparation" (76.0%) were lower compared to the other topics.In the Claude group, the correct response rate reached its peak at 100.0% across four subheadings: "Risk Factors and Complications," "Special Conditions and High-Risk Patient Groups," "Medications and Their Effects," and "Postoperative Monitoring and Discharge." The correct response rates in "General Information on Sedation and General Anesthesia" (84.7%) and "Patient Evaluation and Preparation" (76.0%) were lower in comparison to these peak scores.In the Oral and Maxillofacial Surgeon group, the highest correct response rates were achieved in "Risk Factors and Complications" (88.5%) and "Special Conditions and High-Risk Patient Groups" (87.5%). Comparable rates were observed in "Patient Evaluation and Preparation" (84.4%) and "Postoperative Monitoring and Discharge" (82.3%). The correct response rates in "General Information on Sedation and General Anesthesia" (67.4%) and "Medications and Their Effects" (66.7%) were significantly lower than those of the other topics.In the Anesthesiologist group, the highest correct response rate was determined in "Medications and Their Effects" at 95.8%. Similar and high rates were noted in "Risk Factors and Complications" (89.6%), "Patient Evaluation and Preparation" (88.5%), and "Postoperative Monitoring and Discharge" (88.5%). The correct response rates were relatively lower in "Special Conditions and High-Risk Patient Groups" (85.4%) and "General Information on Sedation and General Anesthesia" (78.5%).In the Pediatric Dentist group, the highest correct response rates were obtained in "Special Conditions and High-Risk Patient Groups" (93.3%) and "Risk Factors and Complications" (91.3%). The correct response rates in "Patient Evaluation and Preparation," "Medications and Their Effects," and "Postoperative Monitoring and Discharge" were identical and at a moderate level of 77.9%. The correct response rate in "General Information on Sedation and General Anesthesia" was the lowest compared to all other topics at 73.1%.

**Table 1: Distribution and Comparison of Knowledge Scores by Study Groups**

| Clinical domains | Chatgpt 4.0 mini (mean ± sd) | Gemini 1.5 pro (mean ± sd) | Claude 3 sonnet (mean ± sd) | Oral & maxillofacial surgeons (mean ± sd) | Anesthesiology specialists (mean ± sd) | Pediatric dentistry specialists (mean ± sd) | Test stat. (h) | p |
| --- | --- | --- | --- | --- | --- | --- | --- | --- |
| General ınfo on sedation & ga | **4.83 ± 0.70** | **5.67 ± 0.56** | **5.08 ± 0.41** | **4.04 ± 1.04** | **4.71 ± 0.81** | **4.38 ± 0.90** | **47.079** | **<0.001*** |
| Patient evaluation & prep | **1.46 ± 0.59** | **3.04 ± 0.69** | **3.04 ± 0.36** | **3.38 ± 0.77** | **3.54 ± 0.83** | **3.12 ± 0.95** | **60.515** | **<0.001*** |
| Risk factors & complications | **4.00 ± 0.00** | **3.79 ± 0.41** | **4.00 ± 0.00** | **3.54 ± 0.59** | **3.58 ± 0.58** | **3.65 ± 0.63** | **22.894** | **<0.001*** |
| Special cons. & high-risk groups | **3.25 ± 0.68** | **3.96 ± 0.20** | **4.00 ± 0.00** | **3.50 ± 0.66** | **3.42 ± 0.72** | **3.73 ± 0.53** | **34.767** | **<0.001*** |
| Drugs used and their effects | **4.00 ± 0.00** | **4.00 ± 0.00** | **4.00 ± 0.00** | **2.67 ± 0.82** | **3.83 ± 0.38** | **3.12 ± 0.65** | **86.617** | **<0.001*** |
| Postoperative monitoring & discharge | **3.88 ± 0.34** | **3.38 ± 0.65** | **4.00 ± 0.00** | **3.29 ± 0.69** | **3.54 ± 0.51** | **3.12 ± 0.59** | **43.036** | **<0.001*** |
| Total score | **21.42 ± 1.02** | **23.83 ± 1.01** | **24.13 ± 0.54** | **20.42 ± 2.69** | **22.63 ± 1.81** | **21.12 ± 2.14** | **66.472** | **<0.001*** |

****p < 0.05 (Kruskal-Wallis Test). SD: Standard Deviation. Total score is based on a maximum of 24 points.***

**Table 2: Comparison of Correct Response Rates Between Groups**

| Knowledge domains | Study groups | Incorrect n (%) | Correct n (%) | Test stat. | P-value | Effect size |
| --- | --- | --- | --- | --- | --- | --- |
| General ınformation on sedation and general anesthesia | **ChatGPT 4.0 Mini** | **28 (19.4%)** | **116 (80.6%)** | **39.545** | **<0.001*** | **0.212** |
|  | **Gemini** | **8 (5.6%)** | **136 (94.4%)** |  |  |  |
|  | **Claude** | **22 (15.3%)** | **122 (84.7%)** |  |  |  |
|  | **Oral & Maxillofacial Surgeon** | **47 (32.6%)** | **97 (67.4%)** |  |  |  |
|  | **Anesthesiology Specialist** | **31 (21.5%)** | **113 (78.5%)** |  |  |  |
|  | **Pediatric Dentistry Specialist** | **42 (26.9%)** | **114 (73.1%)** |  |  |  |
| Patient assessment and preparation | **ChatGPT 4.0 Mini** | **61 (63.5%)** | **35 (36.5%)** | **85.819** | **<0.001*** | **0.383** |
|  | **Gemini** | **23 (24.0%)** | **73 (76.0%)** |  |  |  |
|  | **Claude** | **23 (24.0%)** | **73 (76.0%)** |  |  |  |
|  | **Oral & Maxillofacial Surgeon** | **15 (15.6%)** | **81 (84.4%)** |  |  |  |
|  | **Anesthesiology Specialist** | **11 (11.5%)** | **85 (88.5%)** |  |  |  |
|  | **Pediatric Dentistry Specialist** | **23 (22.1%)** | **81 (77.9%)** |  |  |  |
| Risk factors and complications | **ChatGPT 4.0 Mini** | **0 (0.0%)** | **96 (100.0%)** | **22.076** | **<0.001*** | **0.194** |
|  | **Gemini** | **5 (5.2%)** | **91 (94.8%)** |  |  |  |
|  | **Claude** | **0 (0.0%)** | **96 (100.0%)** |  |  |  |
|  | **Oral & Maxillofacial Surgeon** | **11 (11.5%)** | **85 (88.5%)** |  |  |  |
|  | **Anesthesiology Specialist** | **10 (10.4%)** | **86 (89.6%)** |  |  |  |
|  | **Pediatric Dentistry Specialist** | **9 (8.7%)** | **95 (91.3%)** |  |  |  |
| Special conditions and risky groups | **ChatGPT 4.0 Mini** | **18 (18.8%)** | **78 (81.3%)** | **34.127** | **<0.001*** | **0.242** |
|  | **Gemini** | **1 (1.0%)** | **95 (99.0%)** |  |  |  |
|  | **Claude** | **0 (0.0%)** | **96 (100.0%)** |  |  |  |
|  | **Oral & Maxillofacial Surgeon** | **12 (12.5%)** | **84 (87.5%)** |  |  |  |
|  | **Anesthesiology Specialist** | **14 (14.6%)** | **82 (85.4%)** |  |  |  |
|  | **Pediatric Dentistry Specialist** | **7 (6.7%)** | **97 (93.3%)** |  |  |  |
| Drugs used and their effects | **ChatGPT 4.0 Mini** | **0 (0.0%)** | **96 (100.0%)** | **109.658** | **<0.001*** | **0.433** |
|  | **Gemini** | **0 (0.0%)** | **96 (100.0%)** |  |  |  |
|  | **Claude** | **0 (0.0%)** | **96 (100.0%)** |  |  |  |
|  | **Oral & Maxillofacial Surgeon** | **32 (33.3%)** | **64 (66.7%)** |  |  |  |
|  | **Anesthesiology Specialist** | **4 (4.2%)** | **92 (95.8%)** |  |  |  |
|  | **Pediatric Dentistry Specialist** | **23 (22.1%)** | **81 (77.9%)** |  |  |  |
| Postoperative monitoring & discharge | **ChatGPT 4.0 Mini** | **3 (3.1%)** | **93 (96.9%)** | **34.960** | **<0.001*** | **0.245** |
|  | **Gemini** | **15 (15.6%)** | **81 (84.4%)** |  |  |  |
|  | **Claude** | **0 (0.0%)** | **96 (100.0%)** |  |  |  |
|  | **Oral & Maxillofacial Surgeon** | **17 (17.7%)** | **79 (82.3%)** |  |  |  |
|  | **Anesthesiology Specialist** | **11 (11.5%)** | **85 (88.5%)** |  |  |  |
|  | **Pediatric Dentistry Specialist** | **23 (22.1%)** | **81 (77.9%)** |  |  |  |
| Total score | **ChatGPT 4.0 Mini** | **110 (17.6%)** | **514 (82.4%)** | **86.650** | **<0.001*** | **0.151** |
|  | **Gemini** | **52 (8.3%)** | **572 (91.7%)** |  |  |  |
|  | **Claude** | **45 (7.2%)** | **579 (92.8%)** |  |  |  |
|  | **Oral & Maxillofacial Surgeon** | **134 (21.5%)** | **490 (78.5%)** |  |  |  |
|  | **Anesthesiology Specialist** | **81 (13.0%)** | **543 (87.0%)** |  |  |  |
|  | **Pediatric Dentistry Specialist** | **127 (18.8%)** | **549 (81.2%)** |  |  |  |

***p < 0.05; %: Row percentage; %C: Column percentage. Lowercase letters indicate significant differences between column proportions within a row, while uppercase letters indicate significant differences between row proportions within a column.**
